# Bulletin of the Buffalo General Hospital

**Published:** 1911-07

**Authors:** 


### Bulletin of the Buffalo General Hospital

As one of the first of Buffalo's organised charities the General Hospital has not only a special claim upon the community which it has served so long, but special duties toward it. This bulletin, which has been authorised by the Board of Trustees, and of which a preliminary number appeared in the last issue of the JOURNAL, aims to report, in some measure, the widening scope of the Hospital and to inform the public of the degree in which the institution which it has fostered puts the best medical science of the day at its service. The Bulletin will hereafter be issued quarterly or as occasion may demand.

---

### The Maternity Ward

THE fund for free beds for maternity work, started a little over a year ago, has resulted in a steadily increasing number of maternity cases. From October 1, 1908 to September 1, 1909—the fiscal year of the hospital—the number of such cases was 66. From June 1, 1910, when the new service may be said to have been fairly under way, to June 1, 1911, the maternity cases numbered 149—more than double the previous record. There is reason to expect that this last number will double in the next twelvemonth. Of these 149 cases 109 were on the endowed beds. The donors of those beds have rendered the community a service of the highest value. They have created the opportunity to teach obstetrics clinically. The members of the senior medical class have now proper instruction in a very important and hitherto very much neglected branch of medicine and the internes in charge of the obstetric wards have had a large and valuable experience. Many have been in attendance on from forty to sixty cases. Thirty-two nurses have each seen from twelve to fifteen cases. In addition to the internes Drs. Frank Ransom and William T. Gettman have supervised the deliveries and in especially interesting or difficult cases Drs. Schroeter, Goldsborough and Van Peyma have been in attendance. The patients have not only had better care than they would have received at their homes but they have become acquainted through practical experience with unimagined standards of cleanliness. They receive also a primary education in asepsis or surgical cleanliness which, for its novelty alone, is talked over later with the neighbors. Each mother before leaving the hospital is instructed in the care of her child. The patients' opinion of the service is shown by the fact that they are recommending it to their friends.

One of the semi-circular wards is devoted to the maternity work, with a room for deliveries. In a screened-off portion of the ward are the babies, each carefully tagged, maternal instinct sometimes requiring this reinforcement.

---

Mr. George R. Howard has been elected first vice-president of the Board of Trustees.

Mr. Arnold Watson has been chosen to fill the place of his father, the late Henry M. Watson, who was for eighteen years a member of the Board of Trustees.

On the first of July, Dr. Ross takes possession of his new residence, on the southwest corner of Linwood Avenue and Ferry Street. The superintendent's house on the hospital grounds is to be sold and removed. Its present site will then be improved, in accordance with a general plan for the development of the hospital grounds.

---

### The Radiological Department

It has been the good fortune of those interested in the Hospital, to see during the past year many additions to the working equipment which have largely increased the efficiency of the institution and its staff. Among others, the department of radiology has been enlarged and improved, and there is now available an apparatus for *x*-ray diagnosis, of the most modern and approved type.

This particular field has been extended of late and the department now is a most important factor in the conduct of any first-class hospital.

This method of diagnosis has long been of the greatest value to surgeons in the investigation of injuries and diseases of the osseous system and in the location of foreign bodies as bullets, needles, and the like, that have found accidental entrance into the human economy. Now through the perfection of details in the operative technic, and in the apparatus itself, we are enabled to extend our researches into chest and abdominal conditions, and to approach more nearly a state of accurate knowledge in a given case, a result much to be desired.

While skill and experience on the part of the personnel of the department is necessary, much depends on the apparatus at hand. In the new equipment recently supplied by the hospital little is left to be desired, and the hospital may now call for, and expect to get, as good *x*-ray work as can be found anywhere.

This new instrument is capable of producing a current of the proper sort of such intensity that with correct handling a radiograph may be made of the adult pelvis in less than one second, or what may be termed a snap shot radiograph. The outfit consists of three units—transformer, control stand, and operating table. The two latter are of conventional design planned to facilitate rapid and safe work and have proved their worth in actual use. The chief unit is the transformer, a very good example of an ingenious and well built electrical machine. A five horsepower rotary convertor is the actuating element and, in combination with an open type transformer and rectifying switch, produces a current of sufficient heat and pressure to excite an *x*-ray tube to the degree necessary to obtain skiagrams under the most difficult conditions in the shortest possible time.

The radiological department is in general use by all other departments of the hospital and a rough estimate would place the number of examinations at about twelve hundred during the year. New methods have increased the demands in this direction, and the expense of upkeep has likewise risen. Farsighted executives have met this problem with a policy of generous appropriations and thanks to their attitude this important function of the hospital is now being carried on in an efficient, scientific and systematic manner. The department is in charge of Dr. Plummer and Dr. Schreiner.

---

### The Training School

THE graduation exercises took place Monday evening, June 12, under most favorable conditions. The address of the evening was given by the Reverend William H. Boocock. Dr. James W. Putnam presented the badges and Mr. C. W. Pardee the diplomas. An inspiring feature of the evening was the administering of the Florence Nightingale Pledge by Mr. Pardee, the graduating class standing during its recital in witness that they accepted it.

The entire class with a number of former graduates took the Regents' examinations in June to qualify as registered nurses.

This class illustrates well the differentiation which is creeping into the field of nursing; several members intend to take up institutional work, one will devote herself to the care of children and another whose preference is for obstetric cases hopes to return to the Buffalo General Hospital for further training in that direction.

At the time of graduation seven of the class had enrolled for Red Cross work and most of the others intended to do so. For this service nurses from registered schools only are eligible, and they must have the official endorsement of their school or of some authorised association of nurses. The Red Cross Society desires a large enrollment as, except in case of war, nurses are not required to respond to the call of the Red Cross if it interferes with other work. Nurses who are not Americans, if called on for war service, take an oath of allegiance to the United

States. The General Hospital is well represented on the Red Cross lists.

Some changes in the training school are under way. The system of twelve-hour duty for graduate nurses on special duty in the Hospital went into effect July 1, such nurse to receive twenty-one dollars a week or three dollars a day. This does away with the nurse who, at twenty-five dollars or more a week, was on duty or "on call" for the full twenty-four hours. It has been objected that this change means greater expense for the patient, but such instances will be exceptional. For ordinary cases, the twelve hour service will suffice; while for a patient in a critical condition requiring constant attention, two nurses are always necessary. The new arrangement lightens even that expense when it is inevitable. The training school will keep a list of nurses who desire hospital work, but this will in no way interfere with the present arrangement for registering nurses for private duty, nor will the Training School be limited to its own list. Calls must frequently be made upon the registry as heretofore in order to meet the demands. It is further arranged now that all special nurses are engaged by the hospital, instead of by the patient directly or by his physician. While this leaves both patient and physician as free to choose their nurse as they have been in the past, it brings the nurse herself into a better relation with the hospital.

Probationers will in the future be received in March and September instead of four times a year. This permits improvements in the curriculum as the time which must now be devoted to class work for probationers will be diminished one-half. Probationers will probably be accepted about the fourth month and will be in full uniform before the arrival of the next class. While the Buffalo General Hospital has perhaps had less difficulty than many hospitals in securing a sufficient number of desirable applications, it is a deplorable fact that many well qualified young women cannot take up the work because of lack of means. Uniforms and textbooks are provided, but the fact that there is no remuneration during the three years of study shuts out much good material. A loan fund to meet the needs of such women has been started by one generous gift and when the terms under which such a fund will be administered have been defined, it is hoped that material additions to it will be received. Such gifts would share the distinction of the benefactions which have made possible the maternity service where comparatively small amounts applied at a strategic point have yielded results of high value.

